# Vector saliva controlled inflammatory response of the host may represent the Achilles heel during pathogen transmission

**DOI:** 10.1590/1678-9199-JVATITD-2020-0155

**Published:** 2021-05-17

**Authors:** Claudia Demarta-Gatsi, Salah Mécheri

**Affiliations:** 1 Institut Pasteur, Unité de Biologie des Interactions Hôte Parasites, Paris, France.; 2 CNRS ERL9195, Paris, France.; 3 INSERM U1201, Paris, France.; 4 Medicines for Malaria Venture (MMV), Geneva, Switzerland.

**Keywords:** Vector saliva, Immunomodulation, Parasites, Arboviruses, Vaccine

## Abstract

Infection with vector-borne pathogens starts with the inoculation of these pathogens during blood feeding. In endemic regions, the population is regularly bitten by naive vectors, implicating a permanent stimulation of the immune system by the vector saliva itself (pre-immune context). Comparatively, the number of bites received by exposed individuals from non-infected vectors is much higher than the bites from infected ones. Therefore, vector saliva and the immunological response in the skin may play an important role, so far underestimated, in the establishment of anti-pathogen immunity in endemic areas. Hence, the parasite biology and the disease pathogenesis in “saliva-primed” and “saliva-unprimed” individuals must be different. This integrated view on how the pathogen evolves within the host together with vector salivary components, which are known to be endowed with a variety of pharmacological and immunological properties, must remain the focus of any investigational study dealing with vector-borne diseases.

Considering this three-way partnership, the host skin (immune system), the pathogen, and the vector saliva, the approach that consists in the validation of vector saliva as a source of molecular entities with anti-disease vaccine potential has been recently a subject of active and fruitful investigation. As an example, the vaccination with maxadilan, a potent vasodilator peptide extracted from the saliva of the sand fly *Lutzomyia longipalpis*, was able to protect against infection with various leishmanial parasites. More interestingly, a universal mosquito saliva vaccine that may potentially protect against a range of mosquito-borne infections including malaria, dengue, Zika, chikungunya and yellow fever. In this review, we highlight the key role played by the immunobiology of vector saliva in shaping the outcome of vector-borne diseases and discuss the value of studying diseases in the light of intimate cross talk among the pathogen, the vector saliva, and the host immune mechanisms.

## Introduction

Infectious diseases are the world's most leading cause of death among children and young adults, particularly in low-income countries. They account for 29 out the 96 underlying causes of premature death in humans listed by the World Health Organization (WHO) with roughly 4 million deaths in 2016 [1, 2]. Moreover, epidemiological studies estimate that about 61% of the total number of human infectious diseases are zoonotic [3], while 75% of new diseases discovered in the last decade are zoonotic [4]. From the Greek words "zoon" = animal and "noso" = disease, the zoonotic diseases are infectious diseases that can be transmitted directly or indirectly between animal species and humans. They are caused by harmful germs, including bacteria, parasites, fungi, viruses and prions [5]. Zoonotic diseases can occur via different means, directly or indirectly, by consumption of contaminated food or transmitted via numerous vectors. 

Although they have been recognized for many centuries, in the last years, with increasing levels of contact between humans and wildlife, there has been a significant socioeconomic impact of zoonotic pathogens transmitted from animals to humans worldwide [6], and despite the fact that WHO recommend vaccinations for various zoonotic diseases, they remain a major public health issue worldwide. Additionally, the vector-borne diseases (VBDs), estimated at about 17% of all infectious diseases, represent a fairly good proportion of the neglected tropical diseases (NTDs) in many regions of the world, where they more severely affect the poorest and most vulnerable populations. They are causing more than 700,000 deaths annually and for the most of them there is no vaccine that allows protection, such as malaria, dengue, Zika and leishmaniasis [7]. It is expected that the impact and the prevalence of these diseases will increase substantially in the future with the blooming of a wide array of mosquito species that flourish with the climate change [8, 9]. Vectors play an active role during disease transmission, as disease courses are more severe. Accordingly, delivery of arboviruses in combination with uninfected mosquito bites causes as more severe disease as when viruses are delivered via infectious mosquito bites [10-14]. In mouse models, mosquito and sand fly saliva have also been shown to enhance infectivity and disease progression [15, 16]. From the perspective of infectious diseases, vectors are living organisms, in addition for being themselves infected, that spread infectious agents between organisms of different species. Different populations of a vector species may not exhibit the same ability to transmit a pathogen [17-19]. Many of the vectors are bloodsucking arthropods, such as mosquitoes, ticks and sandflies, which ingest disease-producing microorganisms during a blood meal taken from an infected host (human or animal) and later inoculate it into a new host during their subsequent blood meal. In their quest for a blood meal, vectors transmit pathogens altogether with a cocktail of bioactive molecules present in their saliva into their vertebrate hosts. They can transmit infectious diseases either actively or passively: (i) certain biological vectors (e.g. mosquitoes and ticks) carry pathogens able to multiply within their bodies and are readily delivered to new hosts and (ii) mechanical vectors (e.g. flies) which pick up infectious agents on the outside of their bodies and transmit them through physical contact. Transmission depends on the vector competence and the capacity of the pathogen to cross the various barriers in the vector. These can be influenced by different parameters such as environmental factors (temperature, mosquito midgut microbiome), genetic factors (parasites and hosts), and physiological factors (hosts and parasites) [20, 21]. Moreover, they can be influenced by the specific species of vector involved in the transmission cycle of the pathogen. In fact, in the last decades, with the increase of human movement we assisted to the spread of the main vector such as the *Culicine* mosquitoes responsible of different arbovirus infections, from Africa to the New World. The introduction of competent vector species and pathogenic arboviruses into new geographic regions, where immunologically naïve hosts are present, have profoundly changed the epidemiology of arboviruses. The relevance to geographic distribution is the effect of the environment on both the biology of the vectors but also the relationships between the vectors and the viruses [22]. 

Climatic factors that influence temperature and rainfall, either in intensity, duration or variability, greatly affect the vector population, and consequently, the pattern and level of pathogen transmission and disease propagation [23, 24]. Insects are cold-blooded or poikilothermic organisms, which cannot regulate their own temperature. Since specific body temperatures need to be reached to achieve essential biochemical reactions, the development and physiological functions of the insect is dependent upon the ambient temperature and requires a certain amount of heat to be completed [25]. In fact, at higher temperatures, the mosquito life cycle is shorter than at lower temperatures, and typically there is a species-specific lower temperature threshold at which the species cannot survive [26, 27]. Additionally, the temperature is an important factor to determine the vector competence. In fact, it influences the kinetics of replication and dissemination of viruses and parasites in the vector [28]. Another important climate factor is the frequency and intensity of the rainfalls. It was demonstrated that the vectorial capacity is a function of vector density, which is strongly related to rainfall patterns in the case of mosquitoes [29]. In fact, it has been observed that extreme rainfall followed by floods and increased formation of rain pools have an impact on diseases transmission as these phenomena contribute to the expansion of the vector population. 

Like the human saliva, which is essential for proper functioning of the human body by fulfilling numerous important functions, such as protection against microorganism or disinfection, a prominent function of vector saliva is intimately associated with pathogen transmission. The only tissue of the body where the vector and its saliva, the pathogen, and the vertebrate host immune system are present at the same moment is the skin. Therefore, the skin represents the first barrier against invading pathogens and various antigens and allergens and consists of a complex cellular network that subsequently shapes the systemic immune response. Hematophagy has evolved in parallel with the diversification of salivary constituents to achieve successful blood meal acquisition and to prevent skin defense mechanisms such as hemostasis, pain, itch, and immune effector mechanisms [30, 31]. The saliva of arthropods is widely known to promote and accelerate transmission of pathogens [32, 33]. A comprehensive understanding of the importance of arthropod vector saliva can help shed light on vector-host-pathogen relationship and how these parasites overcome host defenses, revealing new molecules of potential use for control and therapeutic applications. Mosquito saliva is a complex mixture of proteins that allows the mosquito to acquire a blood meal from its host (necessary for egg maturation), by circumventing vasoconstriction, platelet aggregation, coagulation, and inflammation or hemostasis [34]. Moreover, it is well known that mosquito saliva contains proteins that are immunogenic to humans, and some allergic responses can be severe [35, 36]. Recently, the immunomodulatory role of saliva against arboviruses [37, 38] and protozoa including *Leishmania* [33, 39], *Trypanosoma* [40], and *Plasmodium* [16, 36] has been reported. Additionally, because mosquito saliva can be immunogenic, it is speculated it may enhance the pathogenicity by manipulating the host's immune response. The administration of pathogens with vector saliva and their delivery in the skin call for a thorough investigation of immune mechanisms occurring at this site which may influence the outcome of infection. In this review, we discuss the essential role of vector saliva in pathogen transmission, with the focus on malaria parasites, arboviruses and *Leishmania* and highlight the value of considering vector salivary components as possible vaccine candidates against pathogens. 

## Pro-inflammatory and immunomodulatory properties of arthropod saliva 

The human immune system is a network of cells able to discriminate between self and non-self and to mount a response to an invading pathogen, toxin, or allergen, protecting the body against diseases. The host uses both innate natural (killer cells, neutrophils, monocytes, macrophages, mast cells, and dendritic cells) and adaptive (T and B cells) mechanisms to detect and eliminate pathogenic microbes. Once activated, the first step of response constitutes the innate immune response. At this stage, cells produce cytokines and chemokines, which enhance the killing via cytotoxic molecules, and the pathogen phagocytosis, which facilitates pathogen elimination allowing dedicated cells to process antigens for presentation to T cells and subsequent B cell proliferation. At this point, the second step of responses that constitutes the adaptive immunity is initiated by activating and differentiating T and B cells into effector or long-lived antigen-specific memory cells. A major challenge in understanding the pathophysiology of VBDs is not only to decipher the immunobiology of the pathogen but also to characterize the immune-modulatory properties of vector salivary components. 

**Figure 1:**
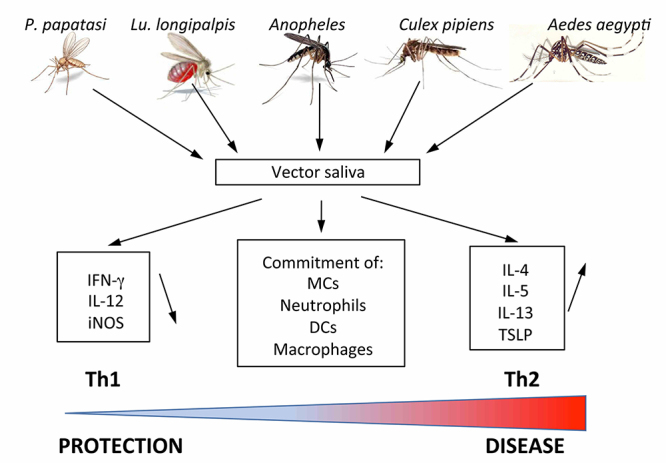
Arthropod saliva has a profound effect on pathogen transmission and on the exacerbation of the disease. Saliva or salivary products from various vectors operate at different levels. They promote the development of a predominant Th2 response, shifting the host response from protection to disease, and they alter the cellular distribution and function of various leukocytes at the bite site.

Athropods saliva is a highly diverse mixture of proteins that can differ among different species [41, 42], among populations originating from different geographical locations [41, 43-45] and between females which feed on blood and non-blood feeding males [41]. Several of these proteins have unknown functions, that allows female arthropods to feed on mammalian hosts, by preventing vasoconstriction [46-48], inhibiting platelet aggregation [49-51], inhibiting blood coagulation cascade [49], and impairing the classical complement pathway [52]. Consequently, all these biological functions impair the capacity of the hemostatic system promoting blood feeding. Additionally, some of these salivary proteins released into the bite site are immunogenic to humans resulting occasionally in severe allergic responses [35, 36], which may facilitate the establishment of infections by manipulating the host immune system (Figure 1). 

## Immunomodulatory effects of mosquito saliva 

Several mouse studies showed that mosquito saliva impairs the frequencies of several immune cells in different tissues promoting the development of a Th2 immune response [38]. Typically, this was the case of two key arbovirus vectors, *Culex pipiens* and *Aedes aegypti*, which had a profound T cell modulatory effect in a virus susceptible mouse model by down regulating and up regulating IFN-γ, and IL-4, respectively, which was not the case in the flavivirus resistant hosts [53]. Moreover, the achykinins sialokinin-I and sialokinin-II, which are present in the saliva of *Aedes aegypti*, mimicked the effect of mosquito feeding by modulating the host T cell responses in the same way [53]. This causal link is not always obvious since recent studies suggest that aggravation of infection by mosquito bites takes place earlier before the adaptive immunity in naive mice occurs, and therefore has no impact on this process. Indeed, a characteristic bite-associated severe infection was observed in severe combined immunodeficiency (SCID) mice, which lack T and B cells, whereas conventional Th1 or Th2 cytokines (e.g., IFN-γ, IL-4) were barely present after mosquito biting of naive wild-type mice in the absence of virus infection [54]. In particular, it was concluded from this study that mosquito bite facilitation of virus infection does not rely on host cutaneous IFN-γ and does not require adaptive immunity. Rather than suppressing or subverting skin anti-viral immune responses, mosquito bites triggered IL-1β?producing inflammatory neutrophils required for the induction of cutaneous inflammatory responses that enhanced Semliki Forest Virus infection [53].

Recently, a study involving human peripheral blood mononuclear cells (PBMCs) engrafted humanized mouse models showed that mosquito saliva affects cytokine levels, increasing anti-inflammatory cytokine production and thus promoting a Th2 immune response after one week post-bite [55]. Classically, a Th2 immune response is a characteristic of parasitic infections or allergen exposure, and tends to dampen inflammatory and cytotoxic responses, both of which are needed to clear parasitic or viral infections. Thus, the mosquito saliva allows the survival of parasites and viruses by triggering a Th2 response instead of a Th1 response. However, other studies pointed out that *Aedes aegypti* mosquito saliva can increase some subsets of immune cells typically associated with a Th1 immune response and generates both Th1 and Th2 response [56, 57] (Figure 1).

The ability of dendritic cells (DCs) to instruct the polarization of naïve T cells into Th1, Th2 or regulatory T cells is intimately associated with the signals that they receive in the peripheral tissues at the time of antigen capture. Thymic stromal lymphopoietin (TSLP), which is a master regulator of allergic inflammation in the skin [58, 59], is produced by epithelial cells, keratinocytes and mast cells (MCs), and is critical in modulating DC function. Enhancement of inflammation-driven TSLP expression results in the influx of eosinophils, neutrophils, and MCs subsequent to macrophage activation, DC maturation, and induction of Th2 cells, leading to pathological expression. DC activation upon TSLP promotes the development of inflammatory Th2 cells that produce the conventional Th2 cytokines IL-4, IL-5, and IL-13 and high amounts of TNF-α. During the sensitization phase of the delayed type hypersensitivity (DTH) response, DCs capture the antigen, translocate to draining lymph nodes and undergo a maturation process necessary for the activation of naive T cells. Not only MC produce TSLP, but they also secrete histamine and other inflammatory mediators that may affect DC maturation, which then fail to ultimately elicit full activation of effector T cells. Notably MCs induce the production of IL-10 via histamine [60]. IL-10 is an important regulator of the DTH response [61], limiting the associated inflammation and tissue damage [62]. Lack of IL-10 results in prolonged DTH response and, conversely, high levels dampen the DTH reaction. 

IgE-dependent type I hypersensitivity is an immediate reaction, also designated atopy and allergy, includes atopic dermatitis (AD), rhinoconjunctivitis and asthma. Augmented secretion of Th2 cytokines IL-4, IL-5 and IL-13, promotes B cell class switching to IgE, leading to enhanced production of IgE in response to allergens. IgE bind to MCs and basophil via high-affinity receptors (FcεRI) as well as to low-affinity receptors (FcεRII/CD23) preferentially expressed on B cells, activated macrophages and eosinophils. Aggregation of receptor bound IgE by allergens triggers the release from MCs and basophils of histamine, leukotrienes and peptides attracting neutrophils and eosinophils. By contrast, DTH, a reaction that takes two or three days to develop, is unrelated to antibodies and is rather under the control of T cells and monocytes/macrophages. Peptides generated from antigens, processed by antigen-presenting cells (APCs), such as DCs and macrophages, are associated to the major histocompatibility complex (MHC II) molecules and presented to CD4^+^ Th1 cells. APCs are activated and secrete IL-12, which stimulates the proliferation and activation of CD4^+^ Th1 cells, which produce IL-2 and IFN-γ, inducing a further release of other Th1 cytokines, thus paving the way to the immune response. DTH is a major mechanism of defense against intracellular pathogens, such as mycobacteria, fungi, and some parasites. DTH also occurs in allergic contact dermatitis and in some autoimmune diseases including multiple sclerosis and coeliac disease. 

The induction of specific IgE in response to mosquito saliva has been well documented. Saliva contains pharmacologically active proteins and peptides [32], which elicit the production of IgE and IgG antibodies [63, 64] and cause a localized allergic reaction in the skin, and dermal hypersensitivity reactions [65, 66]. Both immediate and delayed response in humans were shown to be elicited by *Anopheles* (*An.*) *albopictus* salivary gland extracts (SGE) when inoculated intradermally [67]. Mosquito bites thus result in both immediate and delayed local cutaneous reactions [68, 69]. While these distinct hypersensitivity reactions are relevant to the immune response to saliva, recent work suggests that this response is rather complex and that MCs play a major role in these two responses.

In addition to the classical IgE-dependent activation of MCs, alternative means for MC activation exist; inflammatory responses initiated by MCs at skin sites exposed to mosquito bites were evidenced in naive mice, indicating that MCs can be directly activated in the absence of saliva-specific antibodies [70]. The mosquito bite induced a local cellular infiltrate in the skin and increased cellularity of the draining lymph nodes affecting a broad leucocyte pattern including T and B lymphocytes, DCs, neutrophils and monocytes/macrophages, in short, a conventional DTH response [70]. MCs are a source of TNF-α and macrophage inflammatory protein 2 (MIP-2), which are both promoting neutrophil influx and T cell-mediated DTH response [71, 72]. In a model of contact hypersensitivity reaction, increased amounts of MIP-2 were detected only in the presence of MCs and were associated to DC migration [71, 73]. As a consequence of the induction of MIP-2 by mosquito bites, it was observed an upregulation of IL-10 in the draining lymph nodes with subsequent downregulation of T-cell mediated immune responses mediated by IL-10 [36]. Among saliva constituents that could activate skin MC, Histamine Releasing Factor (HRF), a well conserved protein expressed by all eukaryotic cells including all *Plasmodium* parasite species which acts both intracellularly and extracellularly, was also identified as part of salivary components (personal observation). The capacity of mosquito saliva to upregulate IL-10 expression has been documented across a range of mosquito species and appears as a key generalized immune response. 

The upregulation of IL-10 expression after exposure to mosquito saliva has been observed across a range of mosquito species and is thus a key generalized immune response [36, 38]. IL-10 inhibits the synthesis of IFN-γ, IL-2, and TNF-β [74], antagonizes IL-12, downregulates MHC class II expression by monocytes and inhibits antigen presentation by several APCs [73, 75]. Enhanced IL-10 production can thus antagonize T-cell activation with clear consequences for the development of an efficient immune response against any invading pathogens [76]. IL-10 and perhaps other immunosuppressive mediators produced by MCs in response to mosquito saliva likely result in a dysregulated DTH response and subsequent ineffective antigen-specific T-cell responses. This would have a potentially significant impact on any antigen that is present at the time of saliva inoculation.

## Immunomodulatory effects of sand fly saliva 

Sand fly saliva has chemotactic activity on different immune cells, thereby modifying inflammatory processes at the blood-feeding site. In animal studies, a significant macrophage influx was observed after *Lutzomyia (Lu.) longipalpis* salivary gland homogenate injection that was directly correlated with a higher chemokine expression of CC chemokine ligand 2/monocyte chemoattractant protein-1 (CCL2/MCP-1) in BALB/c strain but not in C57BL/6 mice highlighting the importance of the host background [77]. Other studies using another sand fly, *Phlebotomus* (*P.*) *papatasi,* have shown the ability of its saliva to inhibit the secretion of pro-inflammatory cytokines and consequently enhance the production of anti-inflammatory cytokines, thereby dampening macrophage effector functions. In mice, the *P. papatasi* salivary gland lysate inhibits IL-12 and IFN-γ expression, while the expression of IL-4, which may interfere with the development of a protective Th1 response, was up-regulated [78]. Additionally, it was shown that saliva of *P. papatasi* inhibits the ability of IFN-γ to activate macrophages to produce nitric oxide (NO) facilitating parasite survival. This observation was supported by studies that highlighted the role of two small, ethanol-soluble, thermoresistant salivary molecules, 5’AMP and adenosine, in the downregulation of the iNOS gene expression and reduction of NO production through the inhibition of protein phosphatase 1 and protein phosphatase 2A. These two phosphatases being crucial in modulating the signals that facilitate production of NO [79, 80]. 

The translation of these finding into humans was made possible by the study of the natural exposure to the saliva of *P. papatasi* and *Lu. longipalpis*. Natural exposures resulted in increased IL-10 [81], which inhibits the proliferation of lymphocytes producing IFN-γ, IL-6, IL-8 and IL12p40 [82]. Moreover, human DC, neutrophils, and monocytes are affected by exposure to *Lu. longipalpis*saliva, in particular it was observed that neutrophils undergo an apoptotic program in a FasL-mediated caspase-dependent manner [83]. Saliva was found to alter the expression of co-stimulatory molecules in DC, macrophages, and monocytes [82] and to down-regulate the production of TNF and IL-12p40 in LPS-stimulated monocytes [82]. 

Interestingly, the maxadilan (MAX), a vasodilator peptide present in the saliva of the sand fly, was also able to modulate the host immune response. MAX was reported to up-regulate cytokines associated with the Th2 response (IL-10, IL-6, and TGF-β) and to downregulate Th1 response cytokines (IL-12p70, IFN-γ and TNF-α) and NO [84-86]. Moreover, DCs exposed to MAX showed reduced expression of co-stimulatory molecules (CD80 and CD86) and CCR7 expression and increased secretion of type 2 cytokines suggesting that MAX can act not only on the DCs phenotype, but also on their function [87]. In addition, *P. papatasi* and *P. duboscqi* salivary components were shown to inhibit DC ability to present antigens and subsequently block the immune response initiated by the activation of naïve T lymphocytes and their differentiation into specific subtypes [88]. Another immunomodulatory mechanism associated with the sand fly saliva was the sequential production of prostaglandin E2 (PGE2) and IL-10 by DCs resulting in the downregulation of the cell surface MHC class II and CD86 molecules [88]. Moreover, *Lu. longipalpis* saliva was able to induce lipid body formation and PGE2 production in peritoneal macrophages via the ERK-1/2 and PKC-α signaling pathways that are produced in response to inflammatory stimuli contributing to the development of an anti-inflammatory response [89].

## Modulation of the infection outcome by vector saliva - disease examples 

Among a wide range of insect vectors and their associated pathogens, insect saliva was found to consistently enhance pathology, and infection severity [90]. Conversely, prior exposure to non-infectious bites protects against severe infection; repeated exposure to non-infectious bites eventually results in the elicitation of a Th1 response to salivary antigens and in parallel to the pathogen [91, 92]. Creation of a Th1 biased environment rather than a Th2 biased one is apparently critical in dictating the outcome of a subsequent infection [93]. Thus, saliva could be critical in orienting the immune response mounted against involved parasites.

### Saliva of *Plasmodium* parasite-transmitting vectors

Despite the efforts made, malaria, unfortunately, remains one of the greatest burden of humanity today and is the third leading cause of death among infectious diseases after HIV and tuberculosis. Malaria is caused by protozoan parasites belonging to the genus *Plasmodium* that infects humans, birds, reptiles, and other mammals through the intermediary of an infected female *Anopheles* mosquito. Each year around 3.4 billion people, or almost half of the world's population, are exposed to malaria risk, mainly in the intertropical zone [94]. Africa is the continent mostly affected globally, with nearly 90% of deaths, mainly in the sub-Saharan zone where climatic conditions are particularly favorable to the development of *An.* mosquitoes [95, 96]. However, malaria is not limited to Africa. It also occurs in the tropical and subtropical Asia and Latin America. Recently, despite the numerous efforts to eliminate and eradicate malaria, we must face the increase in resistance phenomena associated with synthetic antimalarial compounds [97, 98] and the use of insecticides [99]. Under these conditions, identification and functional characterization of parasite, vector and host key proteins involved in this multi-system disorder are a major challenge of the post-genomic era of*Plasmodium*research.

The main factors related to the intensity of malaria transmission are population density, longevity, behavior and vector efficiency. The vectors responsible for the transmission of human malaria are arthropods belonging to the subfamily of Anophelinae [100] and to the genus of *Anopheles* [101]. Each species of *Anopheles* occupies a geographical area. More than 484 species belonging in the genus *Anopheles* have been identified [102] of which only about sixty ensure with efficiency, the transmission of human plasmodia. Moreover, they can modify their biting and resting behaviors in evolutionary response to the presence of insecticide-treated mosquito nets, indoor application of residual insecticides, or the absence of preferential host in one location [103, 104]. Human malaria infection starts when a female anopheline inoculates the *Plasmodium* parasites into the skin where it encounters the first line of defense of the human body. Increasing evidence from mouse models to natural infections in human populations provide support for considering the immune response to malaria within an allergic context. Saliva and its allergenic nature through direct response by immune effectors in the skin have significant immediate and long-term effects for the outcome of infection by malaria parasites and the development of clinical immunity [105].

It is recognized that the type of the innate immune response developed at the site of sporozoite inoculation plays a significant role in containing *Plasmodium* infection. In malaria mouse models, it was shown that natural mosquito feeding leads to elevated parasitemia and the increase of more severe forms of malaria. These effects occur following deregulation of immune signalling and a reduction in the recruitment of key inflammatory cells into the inoculation site [106]. This deregulation may be associated with the crucial antiparasitic role played by DCs in cutaneous draining lymph nodes where the first wave of the anti-sporozoite effector CD8^+^ T-cells is triggered by DCs after an infectious mosquito bite [107]. Additionally, another key factor is the balance between anti and pro-inflammatory cytokines. In fact, during pathogen infection, early cytokines responses involving IL-4 and IL-10 increase host susceptibility, whereas responses involving IL-12 and IFN-γ are important for resistance. The immunosuppressive role of IL-10, upregulated by saliva, was shown to exacerbate the infection and disease; early IL-10 expression was associated with increased T regulatory cell proliferation, suppression of Th1 cytokines, as well as the increase of the parasitemia and mortality [108]. In conclusion, both the Type 1 hypersensitivity response, as suggested by IL-4 expression plus the defective Type 4 hypersensitivity response abrogated by IL-10 contribute to increased infection severity and compromise the development of an effective immune response. The immunomodulation of the Type 1 and Type 4 hypersensitivity responses by mosquito saliva creates an immunological environment that hastens disease development with subsequent dysfunction of the host immune system. Mouse model studies have revealed much about the immunomodulatory role of saliva and its impact on the outcome of malaria parasite infection. Interestingly, despite using different species of mosquitoes and different parasite species, there are consistent effects suggesting that there exist generalizable phenomena potentially pertinent to human malaria. Extending from mouse models to natural infections in humans living in malaria endemic settings is a necessary but challenging step. 

The complement system is known to act as a vital component of the immune response against invading pathogens. As an example, C1q interacts with its receptors expressed on neutrophils and phagocytic cells and activates these phagocytes to produce reactive oxygen species to attack pathogens. Saliva of Anopheline mosquitoes and other arthropods contains anti-hemostatic and immune-modulator molecules, among which Complement inhibitors, that favor blood feeding and parasite transmission [109]. Considering the presence of complement inhibitors and other immunomodulatory molecules in arthropod’s saliva, multiple pathogens could benefit from their depressant action during transmission by the vectors. In this regard, two proteins belonging to the SG7 family that are capable of inhibiting the alternative pathway have been described [110]. Complement inhibitors not only facilitate blood feeding of vector arthropods but in addition, they represent an effective strategy that parasites utilize to survive in the host. Indeed, *Plasmodium falciparum* parasites express various proteins, among which C1-INH, that effectively play these roles [111]. 

Following the seasonal expansion of the mosquito population with the rains, mosquito bites were found to be strongly positively associated with an increase in parasite density in chronic pre-existing asymptomatic infections [112-115]. Additionally, individual anti-mosquito SGE IgE titers were also found to be strongly positively correlated with anti-parasite IgE titers. This is consistent with the hypothesis that mosquito bites predispose individuals to develop an IgE anti-parasite response, potentially by the orientation of the immune response to a Th2 profile [53]. Such an orientation of the immune response may lead to a reduced Th1 type response resulting in a lower acquisition of asexual parasite-targeting defense mechanisms and thus a more fertile ground for asexual parasite survival. These observations suggest that the mosquito saliva is responsible for an imbalance in the host Th1/Th2 response by inducing an IgE response and a dysfunctional Th1 response. Such a Th1/Th2 imbalance is characteristic of atopy and thus atopic individuals might be expected to respond differentially to mosquito bites, parasitic infection and the immune-modulatory role of saliva. Orientation of the immune response towards a Th2 profile by allergic diseases - such as asthma or AD - would result in a poor Th1 response and thus amplify the effects of saliva and hence the immunological response to infection. 

In a birth cohort of children living in malaria endemic settings, there was an association of asthma and AD with susceptibility to clinical *Plasmodium falciparum* episodes [116]. In particular, children with clinically defined asthma and especially AD showed an allergy-associated risk of malaria with higher parasite burden during symptomatic episodes, suggesting a reduced ability to contain parasite growth and impaired development of acquired immunity that may stem from their imbalanced Th1/Th2 response. Interestingly, only mosquito saliva, a known major local allergen, was found to be a significant risk factor of AD, inducing a specific IgE response at significantly higher titers in individuals with AD. Considering the strong positive correlation between saliva and parasite IgE titers, this result strongly suggests that a Th2 environment is indeed impairing control of the parasite and undermining the development of anti-parasite immunity. 

In conclusion, the early response of sentinel cells, such as DCs and MCs, determines the evolution of the immune response. Saliva provokes a localized allergic reaction in the skin and induces the production of IgE and IgG antibodies. DCs that are primed by saliva to elicit a Th2 phenotype are more susceptible to orienting the immune response toward a Th2 profile when confronted to a bystander antigen. The orientation of the immune response toward a Th1 profile is crucial for immunity to intracellular pathogens, whereas orientation toward a Th2 profile drives immunity to extracellular pathogens and antigens, resulting in class switching, giving rise to IgE-producing B cells. Anti-saliva IgE titers were found to be strongly associated with the occurrence of atopic dermatitis, which was found to reduce the rate of development of clinical immunity in a birth cohort study. Thus, an atopic Th2 terrain, exacerbated by mosquito bites, influences the course of a single parasite infection and the long-term ability to develop immunity against the parasite.

### Saliva of arbovirus-transmitting vectors

The term arbovirus, from the acronym arthropod and borne, includes several families of viruses transmitted to humans by arthropods such as mosquitoes and ticks. All arboviruses have a common feature, an RNA genome that allows them to rapidly adapt to ever-changing host and environmental conditions. The families of viruses, in the current classification, included in the arbovirus group are Flaviviridae, Togaviridae, Bunyaviridae, and Reoviridae [117]. Arboviruses include more than 250 species with ubiquitous distribution, of which at least 80 cause pathologies in humans. Birds are often reservoirs for arboviruses, which are then passed on to horses, other pets, and humans by mosquitoes. These viruses can be transmitted directly to humans from non-human reservoirs, but interhuman transmission can also occur. Most arbovirus diseases are not transmitted by humans, perhaps because typical viremia is inadequate for infecting the arthropod vector, with some exceptions like dengue fever, yellow fever, zika virus infection and chikungunya disease which can be transmitted from person to person by means of mosquitoes [118]. Transmission efficiency depends on how potent the virus traverse the multiple barriers in the vector and the different interactions among vertebrate hosts, vectors, and viruses that can occur on multiple levels and impact transmission patterns and disease pathogenesis [22]. 

The early events of arbovirus infections are important for the survival of the host, with a close relationship between early peripheral virus burden and mortality [119]. The chance of onward transmission and its ability to cause more pronounced disease is increased during natural infection by mosquitoes. Different studies using mouse models showed that mosquito bite-transmitted arboviruses, or viruses accompanied experimentally by mosquito saliva or SGEs, induce more rapid viremia, higher pathogen load, and greater morbidity compared to needle inoculation in the absence of mosquito-derived factors [13, 54, 120-122]. In fact, it was demonstrated that the intradermal inoculation of the Rift Valley fever (RVF) virus in C57BL/6 mice along with mosquito saliva and SGE increased the mortality rates of mice, as well as the virus titers measured in several organs and in the blood [121]. 

More recently, using a mouse model, it was shown that rather than eliciting anti-viral immune responses, mosquito bites triggered a leukocyte inﬂux that facilitated infection by providing new cellular targets for infection [54]. A two-step process was identified; an influx of cutaneous inﬂammatory neutrophils caused by mosquito bites appeared to be essential for the initiation of the innate immune responses to pave the way for the chemokine receptor CCR2-dependent entry of myeloid cells that are permissive to viral infection. Therapeutic blockade of caspase-1 and neutrophil depletion, the key components of the inflammatory response to the bite, reduced leukocyte inﬂux, suppressed viral replication, and increased host survival. Moreover, in the absence of CCR2-mediated inflammatory myeloid cell recruitment, bites were unable to promote virus infection. 

It is well established that mice deficient in IFN-α/β receptor (*Ifnar* ^-/-^) - that are susceptible to intradermal dengue virus (DENV) infection [123] because of the inability of DENV proteins to interfere with IFN signalling in mice - display characteristic features of human disease, such as lethal vascular discharge, and thus are recognized as a model to study dengue pathogenesis. Using this model, it has been established that only inoculation of DENV in the presence of SGE of a female *Ae*. *aegypti* mosquito was able to exacerbate dengue pathogenesis, and viral infection of dermal macrophages and DCs, and amplified neutrophil and monocyte influx to the inoculated skin site. Moreover, SGE was found to contribute to systemic dengue pathogenesis, by disrupting the endothelial barrier function. More interestingly, the removal of the skin site 4h post-inoculation of the virus alone rescued mice from developing severe disease, while no rescue was observed when SGE was present. These data underline the essential role of mosquito-derived products in the rapid spreading of the virus beyond the skin and in enhancing the disease severity [124]. 

Disease pathogenesis promoting capacity of Arthropod saliva was also observed in humans. The innate immune system represents the first barrier against inoculated flaviviruses such as DENV in the skin. A family of pattern recognition receptors (PRRs) including toll like receptors (TLRs), retinoic-acid-inducible gene I (RIG-I), melanoma differentiation-associated gene 5 (MDA5), and protein kinase R (PKR) were found to be induced during DENV infection [125]. Replication of this virus in cultured human keratinocytes was found to be enhanced by *Aedes aegypti* salivary proteins [125] by inhibiting the secretion of antimicrobial peptides (AMPs), S100A7, Elafin, as well as IFNs in the earliest stages of infection [126]. In a more recent study, in contrast to keratinocytes infected with DENV alone, a significant increase in the expression of DENV transcripts was observed in keratinocytes infected with DENV in the presence of salivary proteins, among which the 34-kDa protein. This was associated with a strong suppressive effect on the interferon regulatory factors (IRF3 and IRF7), resulting in the abrogation of type I IFN production. The authors proposed that the identification of the 34-kDa protein in *Aedes aegypti* saliva could serve as a target for the control of DENV replication in vertebrate hosts [127]. 

### Saliva of *Leishmania*-transmitting vectors

*Leishmania* diseases are a group of human zoonotic VBDs caused by an intracellular protozoan parasite of the genus *Leishmania* and inoculated to humans by infectious bites of a female sand fly, essentially of the genera*Phlebotomus*for Old World and*Lutzomyia* for the New World. Leishmaniasis is one of the top three NTDs caused by protozoa representing a serious world health problem with a broad spectrum of clinical manifestations of infection ranging from cutaneous ulcers to a visceral form, with a potentially fatal outcome [128, 129]. The severity of clinical features depend on the species of*Leishmania*involved and on the immune response developed by the host. Worldwide, 1.5 to 2 million new cases occur each year, 350 million are at risk of acquiring the disease, and leishmaniasis causes 70,000 deaths per year [130]. 

In the past years, a consistent progress in diagnostic and therapeutic approaches has significantly affected the management of leishmaniasis. However, leishmaniasis mortality and morbidity sill show an increasing trend worldwide. One of the reasons for that is the large variety of vectors that can transmit the parasites. All the 20 recognized *Leishmania* species that are pathogenic for humans can be transmitted by 100 out of the 900 different species of sand fly recorded [131, 132]. Additionally, it was observed that the phlebotominae potentially implicated in *Leishmania* transmission belong to 13 genera at least [133, 134], assessing the importance of the vector in the endemic setting. As mentioned before, infected vector females feed on mammalian hosts and regurgitate parasites together with the salivary proteins into the bite site and release different proteins endowed with immunomodulatory properties, which facilitate the establishment of the infection. 

The above-mentioned effects of sand ﬂy saliva on the host’s immune systems results in an altered environment at the feeding site that favors the development of *Leishmania* disease in the infected host. Parasite growth enhancement was demonstrated in various inbred strains of mice with variable susceptibility to *Leishmania* infection. In fact, it was shown that the chemotactic effect of saliva, responsible for the increase in the influx of neutrophils and macrophages at the blood-feeding site, is more pronounced when *Leishmania* parasites are co-injected with sand fly salivary molecules [77]. This resultsd in an exacerbated disease reﬂected by a larger ulcer that developed into a necrotic lesion compared to the mice receiving the parasite alone [33, 135, 136]. Additionally, co-inoculation of *P. papatasi* saliva with *L. major* converted the naturally resistant C57BL/6 mouse strain into a non-healing phenotype associated with an early increase in Th2-related cytokines such as IL-4 and IL-5 [33]. Moreover, the same co-inoculation in CBA mouse strain is responsible of an upregulated expression of IL-4 and a reduced production of IFN-γ, IL-12, and iNOS [78], resulting in the promotion parasite proliferation inside the host. Following these observations, a series of *in vitro* studies aiming to explore the mechanisms responsible for the parasite growth in the presence of sand-fly salivary molecules were performed. 

Co-inoculation of*L. amazonensis*with*Luu. longipalpis*saliva was associated with elevated IL-10 production, leading to the suppression of effector functions of monocytes and macrophages [137]. This observation was confirmed in*in vivo*studies following exposure of mice to*L. longipalpis*infected sand fly bites [138], and during stimulation of the peritoneal cavity with*L. major*plus*Lu. longipalpis*saliva [139], in both of which IL-10 production was observed. Additionally, neutrophils play an important role, as the first-recruited host cells to the feeding site, for the parasite survival in the vertebrate host. In fact, they act as a “reservoir” able to protect the promastigotes from a rapid degradation by cytotoxic activity of natural killer cells, neutrophils, and eosinophils in the vertebrate host before they invade macrophages [140]. Moreover, neutrophils incubated with *L. chagasi* and sand flay saliva produced significantly higher amounts of MCP-1 (CCL2) that attracts macrophages for the clearance of these recruited infected neutrophils [83, 141]. Importantly, MAX, mentioned above, was able alone to exacerbate *L. major* infection [142] due to its capacity to upregulate IL-10 and TGF-β production and to suppress IL-12p40, TNF, and NO production [86]. Similar results were observed with other *Leishmania*-sand fly combinations, such as *Lu. Longipalpis-L. braziliensis* [143], *Lu. longipalpis-L. amazonensis* [135], *Lu. longipalpis-L. chagasi* [43], *Lu. longipalpis-L. mexicana* [143], and *Lu. whitmani-L. braziliensis* [144] and *P. duboscqi-L. major* [145]. More important is that the enhancing effect is unique to sand fly saliva since saliva from *An. aegypti*, *Rhodnius prolixus, or Ixodes scapularis* did not enhance *L. major* infectivity in mice [136]. 

## Vector salivary components as vaccine candidates against pathogens 

New interventions, such as drugs and insecticide-treated bed nets became available over the last decades to reduce the burden of infectious diseases. Recently, this progress has been halted due to vector resistance and the emergence of pathogen resistance to treatments. Therefore, it is important to develop new strategies to control and eliminate the zoonotic infectious diseases. Vaccines are among the main defenses against infectious diseases, such as tick-borne encephalitis virus, Japanese encephalitis virus, and YFV. However, the development of effective vaccines is not always successful. One of the most trivial examples is the history of malaria vaccine development where the most established vaccine (RTS,S), a recombinant protein containing regions of the *Plasmodium* circumsporozoite protein (CSP) and targeting the sporozoites stage of the parasite confers less than 40% of protection [146, 147]. 

**Figure 2:**
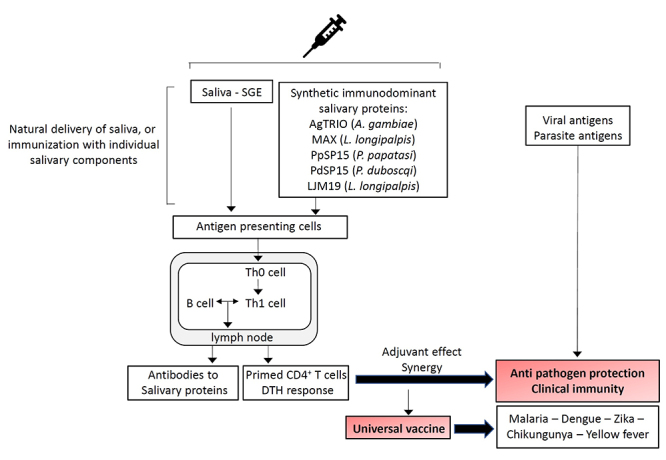
Mosquito and sand fly saliva-based vaccines: proposed mechanism of action. Exposure of the vertebrate hosts to saliva collected from salivary glands or to purified salivary components have the capacity to prime the immune system by eliciting antibodies to salivary components or to induce a delayed type hypersensitivity response (DTH). When the host is exposed to parasite or viral antigens delivered via infectious vector bites, vector saliva may generate an adjuvant effect in the skin for the priming of an anti-pathogen Th1 immune response necessary for protection. The saliva-based vaccine is able to elicit an increase of both anti-saliva and anti-pathogen IgG antibodies and cellular (specific CD4^+^ and CD8^+^ cells) immune responses, resulting in a reduction of pathogen load in the immunized individuals. For vector-borne arboviruses for example, this may lead to a “universal” vaccine derived from mosquito saliva that could be a solution to offer some protection in the emerging setting of an arboviral epidemic.

Given the complexity of the infectious agent’s interactions with the host immune system, vector-based vaccine approaches may offer a solution to control VBD by taking advantage of a common variable the vector saliva. The importance of vector saliva proteins to promote the infectivity of the pathogens carried in the saliva and the establishment of systemic infection can be exploited in a novel vaccine approach through vaccination with arthropod saliva, such as saliva from ticks, sand flies, or mosquitos, conceivably preventing the infection by creating an immune environment that blocks transmission or destroys the pathogen (Figure 2). 

Moreover, by targeting the vector-pathogen-host interface, one can bypass *in vivo* disease-specific manifestations as the vector-based vaccine acts very early at the site of the vector bite in the skin, pre-empting or complementing host anti-pathogen immune responses. In support of this type of vaccine, several studies have demonstrated their efficacy. Studies on the phlebotomine sand fly *Lutzomyia* are more advanced than those dealing with mosquitoes, as more detailed biochemical characterization of salivary components is available. As a consequence, the first studies aimed to test the capacity of the immune response to salivary proteins to protect humans were performed in leishmaniasis field as saliva component of the sand fly *Lutzomyia* is more extensively characterized than other vector-borne saliva. It was initially shown that parasite transmission is more effective in naïve mice than in mice previously exposed to non-infectious bites which developed a strong DTH response with IFN-γ production at the site of parasite inoculation conferring a protective response against *Leishmania major*, suggesting that it is possible to develop a vaccination strategy against saliva proteins [91]. 

Furthermore, different preclinical studies of infection on different animal species offer a proof-of-concept to the vaccine strategy using vector salivary components. Recently, Oliveira at al. identified PdSP15, a salivary protein responsible of the protective effect, by reverse antigen screening of *P. duboscqi* sand fly salivary molecules in saliva-exposed non-human primates [148]. They showed that immunization of rhesus macaques intradermally with PdSP15 DNA and boosted few days later with recombinant PdSP15 (rPdSP15) prevents cutaneous leishmaniasis transmitted by *Leishmania major* infected sand ﬂies. The study demonstrated a correlation between a Th1 cell-mediated immune response and protection with cutaneous appearance of saliva-specific CD4^+^IFN-γ^+^ cells within the DTH site that generates an early *Leishmania*-specific immune response contributing to parasite killing in the dermis and primed specific immunity to the parasite (Figure 2). This can explain the parasite protection acquired after exposure to non-infectious bites. In this case the vector saliva may generate an adjuvant effect in the skin for the priming of a Th1 anti-parasite immune response necessary for protection. The authors also provided evidence that protection mediated by anti-PdSP15-specific immune response was cell-mediated and antibody-independent. 

Additionally, the high homology of the protein between *Leishmania* vectors suggests that PdSP15 may protect against disease transmission in various areas in the world and the possible development of a universal vaccine. This cross-protection was confirmed when mice exposed to *P. papatasi* were subsequently infected with *P. duboscqi* SGE plus *L. major* [149]. The translational relevance of the study was demonstrated by testing the immunogenic capacity of rPdSP15 in endemic area. Sera and PBMCs cells from individuals naturally exposed to *P. duboscqi* bites recognized PdSP15, demonstrating its immunogenicity in humans. Moreover, PdSP15 sequence and structure show no homology to mammalian proteins, further demonstrating its potential as a component of a vaccine for human leishmaniasis. Similarly, immunity to *Lu. longipalpis* saliva LJM19 protein in hamster and to different sand fly salivary proteins in beagles protects against visceral leishmaniasis (VL), underlining the protective role of a Th1 response against the infection and confirming the protective role of salivary proteins against both cutaneous and visceral leishmaniasis [150, 151]. Another study showed that mice vaccinated with the *Lu. longipalpis* salivary component MAX, responsible of the vasodilatation and immunosuppressive and anti-inﬂammatory effects, developed both cellular responses and antibodies against the salivary protein protecting against the infection. Furthermore, in the case of disease transmission by *Lu. longipalpis*, MAX was thought to be the major exacerbative element since vaccinating against this molecule neutralized the effects of whole saliva [142]. 

Recently, a study trying to generate a more affordable and easily manufactured anti-leishmaniosis vaccine demonstrated that the synthetic full length MAX molecule as well as C and N terminal peptides derived thereof can be utilized successfully as antigens in a cationic lipid DNA complex (CLDC) adjuvant vaccine system protecting three different strains of mice (BALB/c, C3H and C57BL/6) against footpad challenges with *Leishmania major* co-injected with MAX. In the protected mice the immune response was characterized by an increase of IFNγ and a decrease of IL-4 secretion from CD4^+^ cells in footpad-draining lymph nodes [152]. This suggests an increased Th1-bias that is potentially capable of protecting against intracellular *L. major* infection. These different studies demonstrated that immunity to salivary component may prevent the reprograming of innate immune responses permitting a more protective host cellular response against parasite transmission, growth, and persistence. This may lead to think that the combination of various *Leishmania spp*. antigens and salivary proteins could provide the best components for an efficacious vaccine. 

Different possible combinations of sand fly saliva or salivary proteins with *Leishmania* antigens or attenuated *Leishmania* parasites were tested demonstrating their effectiveness. The LBSapSal vaccine, proposed as an alternative approach for interrupting the domestic cycle of *Leishmania infantum*, was tested in dogs with the intention of protecting against canine visceral leishmaniasis. Composed of *Leishmania braziliensis* antigens adjuvanted with saponin and *Lutzomya longipalpis* SGE, the vaccine was able to elicit an increase in both anti-saliva and anti-*Leishmania* IgG antibodies and cellular (specific circulating CD8^+^ cells) immune responses resulting in a reduction of splenic parasite load in the immunized groups [153]. Other studies highlighted the fact that vaccine combinations were protective only when animals were first primed with DNA sequence of the salivary protein and then boosted with the vaccine combination. This was first observed when mice primed with the sand fly salivary protein PpSP15 DNA and then boosted with a combination of PpSP15 and live non-pathogenic *L. tarentolae* expressing the cysteine proteases (type I and II, CPA/CPB) displayed better immunity and protection against cutaneous leishmaniasis compared to animals vaccinated with PpSP15 or with the attenuated *L. tarentolae* parasites alone [154]. This result was confirmed by another study where animals vaccinated simultaneously with the *Leishmania* antigen KMP11 and the salivary protein LJM19 showed no improvement in the protective efficacy over the KMP11 or LJM19 vaccines alone [155]. Leishmania vaccine development is advancing in preclinical trials, with at least 3 promising candidates, considering the natural transmission of the parasite and the priming of animal models with sand fly bites before vaccination able to boost the Th1 response [156]. 

In the malaria field, it was recently demonstrated that the hyperimmune antisera prepared against *An. gambiae* SGE partly protected mice from mosquito-transmitted *Plasmodium,* with a decrease of early hepatic stage infection and lower levels of parasitemia when exposed to infected mosquitoes. Using DNA yeast surface display library, they identified the antigens recognized by SGE antiserum that contributed to the diminished levels of *Plasmodium* infection. The screen identified the *An. gambiae* TRIO (AgTRIO) protein, expressed only in salivary gland and not in other organs, with putative signal sequences, suggesting that it is secreted into saliva. Moreover, it was demonstrated that the presence of *Plasmodium berghei* sporozoites in the salivary glands increase the AgTRIO expression [157, 158] and production, and that its depletion does not alter mosquito probing time and blood-feeding behaviour [159]. The protective effect of antibodies against AgTRIO was tested in naïve mice that received AgTRIO antiserum and were challenged with *Plasmodium berghei*-infected *An. gambiae* mosquitoes. The administration of AgTRIO antiserum resulted in a decrease in the parasite burden in the liver and blood stage parasite levels, suggesting that *Plasmodium* sporozoites are directly or indirectly affected by AgTRIO antibodies and are unable to establish a high level of hepatic infection. 

More interestingly, the same study was performed in FRG human liver chimeric mice, which support liver stage infection with *Plasmodium falciparum* [160, 161] and seven days after blood meal with *Plasmodium falciparum*-infected *An. gambiae* or *An. stephensi* mosquitoes, mice who received the AgTRIO antiserum had reduced infection levels compared with the control groups. The study showed that the AgTRIO antiserum diminished the movement of sporozoites in the murine dermis. As the number and the viability of sporozoites that reach the liver is an important factor for the disease development, any impact on this process can greatly alter the initial pathogen burden during systemic infection. In contrast to what was previously shown where exposure to mosquito bites did not protect against malaria infection [162, 163], which could be attributed to several factors, starting from the quantity of saliva inoculated to the host [81] or the short duration of a mosquito bite [162]. It is well known that under natural conditions, some salivary components of mosquitoes induce an antibody response in humans [164-167]. In the study mentioned before, it was shown that individuals or mice exposed to bites of *An. gambiae* presented low IgG responses to AgTRIO, suggesting a natural lack of immunogenicity following mosquito exposure, which allows a severe disease to occur. Finally, the study highlighted the ability of AgTRIO antibodies to enhance the efﬁcacy of CSP antibodies against malaria, suggesting a synergistic efficacy of anti-CSP antibodies and antibodies to salivary components in controlling the infection.

Despite the fact that host immune responses to vector bites may be highly variable in endemic areas, given an individual’s lifelong exposure to certain mosquito species and parasites or viruses they carry, salivary molecules constitute a unique link between a variety of different VBDs [168-170]. For this reason, with the increase of emerging arbovirus infections and the non-availability of an effective vaccine during the epidemic period, the development of a “universal” vaccine derived from mosquito saliva could be a solution to offer some protection in the emerging setting of an arboviral epidemic (Figure 2). Recently, the AGS-v vaccine, a mosquito-borne disease vaccine which rather than targeting specific pathogens, elicits an immune response to four salivary peptides isolated from *An. gambiae* salivary glands, that are shared by several mosquitoes. Hence, the vaccine could potentially protect against numerous mosquito-borne infections including malaria, dengue, zika, chikungunya and yellow fever (Figure 2). Thus the vaccine is the only universal mosquito-borne disease vaccine in Phase I clinical trial in healthy volunteers living in non-endemic areas [171]. 

## Conclusions 

Mosquitoes and other vectors and the diseases they transmit are of growing public health concern. Often, there are no prophylaxis for these diseases other than vector control measures and no cure other than palliative care. Understanding how vector saliva interacts with the human immune system not only helps to understand the mechanisms of the disease pathogenesis but also could provide therapeutic solutions. The shift in the paradigm that vector saliva is more than simply a vehicle for pathogen transmission but rather a fluid endowed with a determining capacity in terms of pathogen virulence has opened new opportunities towards the design of vaccines against VBDs. Having said that, novel composite formulations combining both vector saliva components and pathogen-derived antigens represent another path towards the design of more elaborated and efficient vaccines against numerous VBDs. 

### Abbreviations

AD: atopic dermatitis; AMPs: antimicrobial peptides; *An.*: *Anopheles;* CSP: circumsporozoite protein; DCs: dendritic cells; DENV: Dengue virus; DTH: delayed type hypersensitivity; FcεRI: IgE bind to high-affinity receptors; HRF: histamine releasing factor; *Lu.*: *Lutzomyia;* MAX: maxadilan; MCs: mast cells; MDA5: melanoma differentiation-associated gene 5; NO: nitric oxide; NTDs: neglected tropical diseases; *P.*: *Phlebotomus*; PBMCs: peripheral blood mononuclear cell; PGE2: prostaglandin E2; PKR: protein kinase R; PRRs: pattern recognition receptors; RIG-I: retinoic-acid-inducible gene I; RVF: Rift Valley fever; SCID: severe combined immunodeficiency mice; SGE: salivary gland extracts; TLRs: toll like receptors; TSLP: thymic stromal lymphopoietin; VBDs: vector-borne diseases; VL: visceral leishmaniasis; WHO: World Health Organization.
